# PVL positive methicillin resistant *Staphylococcus aureus* breast abscess infection among post-partum women in Chennai, South India

**DOI:** 10.1186/1471-2334-12-S1-O13

**Published:** 2012-05-04

**Authors:** Nagarajan Abimanyu, K Arunkumar, M Saravanan, G Sivakumar, Padma Krishnan

**Affiliations:** 1 Dept. of Microbiology, Dr. ALM Post Graduate Institute of Basic Medical Sciences, Chennai, India; 2 Madras Medical College and Govt. General Hospital, Chennai, India

## Background

Breast abscess and mastitis caused by MRSA is on the rise. This could result in poor response to routinely used β-lactam antibiotics. Lack of MRSA screening could lead to wrong choice of antibiotic after drainage resulting in multiple and recurrent abscesses leading to increased morbidity and mortality.

## Methods

A total of 20 isolates of *Staphylococcus aureus* were isolated from 25 cases of breast abscess from a tertiary hospital during January 2010-October 2010. Isolation was done by culturing the pus sample drained from the abscess with 16 gauge needle. The *S. aureus* isolates were screened for susceptibility to various antibiotics including cefoxitin, clindamycin, fusidic acid, mupirocin, vancomycin and linezolid. MIC to oxacillin, erythromycin and clindamycin were performed using E-strip method for resistant isolates. Molecular detection of methicillin resistance and Panton-Valentine Leukocidin (PVL) were done by multiplex PCR targeting *fem*A, *mec*A, and lukS-PV.

## Results

Of the 20 isolates, 10 (50%) were found to be MRSA, of which 3 (30%) isolates were found to be positive for PVL. Of the tested isolates, highest sensitivity (100%) was observed for vancomycin, linezolid, mupirocin, clindamycin and fusidic acid. Highest resistance was observed for co-trimoxazole>erythromycin>ofloxacin> netilmicin>gentamicin>amikacin. High-level methicillin resistance (MIC = 256µg/mL) was observed for 3 MRSA isolates from hospitalized patients.

## Conclusion

From the above results, the PVL-MRSA infections in breast abscess among post partum women is found to be increasing. Proper drainage and routine screening of MRSA from breast abscess should be done to decrease the morbidity and mortality.

